# The Diagnostic Challenge of Hypophysitis vs. Non-Functioning Pituitary Macroadenomas: An Updated Review and Comparative Analysis of Distinguishing Criteria

**DOI:** 10.3390/diagnostics16020328

**Published:** 2026-01-20

**Authors:** Taieb Ach, Ines Bouzaouech, Ayoub Gasmi, Nassim ben Haj Slama, Aicha Ghachem, Lamys Abbes, Imen Halloul, Wiem Saafi, Hamza El Fekih, Ghada Saad, Yosra Hasni, Houda El Mhabrech

**Affiliations:** 1 Faculty of Medicine of Sousse, University of Sousse, Sousse 4002, Tunisia; bzw.ines@gmail.com (I.B.); ayoub.gasmi.007@gmail.com (A.G.); nassimbhs@ymail.com (N.b.H.S.); ghachemaycha@gmail.com (A.G.); lamysabbes5@gmail.com (L.A.); imen.halloul@gmail.com (I.H.); wiem.saafi@gmail.com (W.S.); elfekihamza@gmail.com (H.E.F.); ghada.saad6587@gmail.com (G.S.); y.hasni@gmail.com (Y.H.); mhoudafr@yahoo.fr (H.E.M.); 2 Department of Endocrinology, University Hospital of Farhat Hached Sousse, Sousse 4031, Tunisia; 3 Laboratory of Exercise Physiology and Pathophysiology, L.R.19ES09, Sousse 4054, Tunisia; 4 Department of Radiology, University Hospital of Farhat Hached Sousse, Sousse 4031, Tunisia

**Keywords:** hypophysitis, pituitary, macroadenoma, score, diabetes insipidus

## Abstract

**Background:** Differentiating hypophysitis from non-functioning pituitary macroadenomas (NFPMA) remains a clinical and radiological challenge. Both entities present as sellar masses with overlapping features but require distinct therapeutic approaches. Accurate preoperative identification is necessary to avoid unnecessary surgery in inflammatory forms. This review aims to compare the clinical, endocrine, and imaging characteristics of hypophysitis and NFPMA, incorporating recent findings and evaluating the performance of three diagnostic scoring systems currently in use. **Methods:** A comprehensive narrative literature review was conducted using original articles, clinical series, radiological studies, and systematic reviews retrieved from international databases. The analysis focused on demographic characteristics, clinical presentation, hormonal profiles, magnetic resonance imaging (MRI) features, and the comparative evaluation of the three published diagnostic scoring systems designed to differentiate hypophysitis from NFPMA. **Results:** Hypophysitis predominantly affects women, particularly during late pregnancy or the postpartum period, and is frequently associated with autoimmune diseases. Corticotropic deficiency and central diabetes insipidus (CDI) are disproportionately frequent in hypophysitis, whereas somatotropic deficiency is more characteristic of NFPMA. Radiologically, hypophysitis typically appears as a smaller, symmetric, and homogeneous mass with intense, uniform contrast enhancement, associated with pituitary stalk thickening and loss of the posterior pituitary bright spot. In contrast, NFPMA generally present as larger, asymmetric, and heterogeneous lesions, frequently invading the cavernous sinus and compressing the optic chiasm. Analysis of the three diagnostic scores indicates that combining clinical, hormonal, and imaging data improves accuracy compared to relying on single features. The most recent score includes hormonal markers, which significantly enhance sensitivity and specificity, emphasizing the importance of integrated assessment. **Conclusions:** No single clinical, hormonal, or imaging feature is pathognomonic. However, integrating clinical context, endocrine profile, imaging characteristics, and validated diagnostic scores significantly enhances preoperative diagnostic accuracy. The systematic use of composite scores may help optimize therapeutic decision-making and reduce unnecessary surgical interventions in patients with hypophysitis.

## 1. Introduction

The pituitary gland is a small but vital endocrine organ located in the middle cranial fossa, within a bony cavity of the sphenoid bone known as the sella turcica. It consists of two embryologically distinct parts: the anterior lobe (adenohypophysis), composed of several hormone-secreting cell types responsible for the release of trophic hormones, and the posterior lobe (neurohypophysis), which serves as a storage site for oxytocin and vasopressin (antidiuretic hormone, ADH) [1]. Lesions of sellar or parasellar origin encompass a wide spectrum of entities, ranging from common to exceedingly rare disorders [2].

Because of the complex anatomy of the sellar region and the dual embryological origin of the pituitary, a variety of pathologic processes may occur in this area [2,3].

Among these conditions, pituitary adenomas (PA) represent by far the most frequent etiology, accounting for approximately 15% of all intracranial tumors and up to 90% of intrasellar masses [1]. These are benign, slowly growing tumors arising from the hormone-secreting cells of the adenohypophysis, with malignant transformation being exceptionally rare [4]. PA are typically classified according to size: microadenomas (<10 mm) and macroadenomas (≥10 mm), and hormonal activity, distinguishing between functioning pituitary adenomas and non-functioning pituitary adenomas [4,5]. Non-functioning pituitary macroadenomas (NFPMA) are benign tumors originating from the anterior pituitary that do not exhibit biochemical or clinical evidence of hormone hypersecretion [4]. They account for 14 up tp 54% of all PA and are usually discovered through manifestations of mass effect, such as headaches, visual disturbances, or hypopituitarism [2,6]. While functioning macroadenomas are often easily recognized due to their specific hormonal syndromes, NFPMA represent a more subtle diagnostic challenge. Many other non-secreting sellar lesions may mimic their radiologic and clinical features, presenting with a similar pattern of mass effect and visual symptoms [7].

Hypophysitis, an inflammatory condition of the pituitary gland, remains one of the most challenging differential diagnoses among these mimickers [8]. Hypophysitis refers to a heterogeneous group of disorders characterized by inflammatory infiltration of the pituitary parenchyma, with variable etiology and histopathological patterns [9]. Although rare, accounting for less than 1% of all pituitary lesions and 0.5% of cases of hypopituitarism [10,11,12], hypophysitis has been increasingly recognized in recent years, partly due to the widespread use of MRI and the identification of novel immune-related forms associated with immune checkpoint inhibitors. The inflammatory process may involve the anterior pituitary (adenohypophysitis), the posterior pituitary and infundibulum (infundibulo-neurohypophysitis), or both (panhypophysitis) [9]. Primary hypophysitis refers to inflammatory processes that originate within the pituitary gland and remain confined to it, most often attributed to an autoimmune origin [13]. Secondary hypophysitis, in contrast, is triggered by a wide range of local or systemic conditions. Worldwide, immunotherapy has become the most frequent cause of secondary hypophysitis [14,15]. More recently, Severe Acute Respiratory Syndrome Coronavirus 2 (SARS-CoV-2) vaccination has also been reported as a potential trigger [16]. From a histopathological perspective, hypophysitis comprises six subtypes, provided that a pituitary biopsy can be obtained [17,18,19,20,21].

The management of hypophysitis is primarily medical, based on hormonal replacement therapy and corticosteroids, while surgery is reserved for refractory cases or for those presenting with severe compressive symptoms [22,23,24,25,26]. Although biopsy provides the definitive diagnosis, it is not without significant risks, including visual deterioration, central diabetes insipidus (CDI) and permanent pituitary dysfunction [27]. For this reason, accurate preoperative differentiation from other sellar masses, particularly NFPMA, is of utmost importance to avoid unnecessary surgery and its potential complications. In clinical practice, many equivocal sellar lesions are initially suspected to represent NFPMA, underscoring the difficulty of differentiating these tumors from hypophysitis based solely on preoperative evaluation [28,29].

NFPMA are categorized based on the hormone and transcription factor expression revealed on immunohistochemistry (IHC). Tumors that express one or more anterior pituitary hormones or their transcription factors on IHC but without any clinically relevant hypersecretion are designated as silent pituitary adenomas [30,31,32]. Conversely, clinically silent adenomas may show hormonal overproduction on laboratory testing, yet without corresponding clinical manifestations [33,34,35,36]. Despite advances in MRI characterization, no single feature is pathognomonic for either entity, and both conditions most commonly appear as solitary, homogeneous, or mildly heterogeneous sellar masses with varying degrees of stalk deviation, enhancement, and mass effect.

Several studies have sought to refine criteria for distinguishing hypophysitis from NFPMA. Predictive scoring systems, such as those by Gutenberg et al. [37] and Wright et al. [38], while more recent work, including North African studies such as the score proposed by Ach et al. [39], have contributed to this effort.

This review synthesizes clinical, hormonal, and imaging data from diverse sources to clarify diagnostic challenges and propose practical strategies for improving preoperative evaluation.

## 2. Methods

The review was conducted following established standards for narrative syntheses in medical research. A broad literature search was performed across major biomedical databases, including PubMed, Embase, and Cochrane Library, to gather and analyze current evidence on differentiating hypophysitis from NFPMA. Keywords and search phrases included, but were not limited to, “hypophysitis,” “autoimmune hypophysitis,” “pituitary inflammation,” “non-functioning pituitary macroadenoma,” “sellar masses,” “pituitary MRI,” “endocrine insufficiency,” “hormonal deficit,” and “diagnostic score.” Boolean operators (“AND,” “OR”) were used to expand or restrict the search as appropriate, and filters were applied to include only studies conducted in humans and published in English or French.

All retrieved articles were screened by title and abstract to identify those relevant to the diagnostic differentiation between hypophysitis and NFPMA. The reference lists of the selected publications were manually reviewed to identify additional pertinent studies not captured in the initial database search. When available, recently published conference abstracts, review articles, and expert consensus statements were also considered, particularly when they contributed novel insights into imaging or hormonal diagnostic criteria.

Eligible studies included original research articles, both prospective and retrospective clinical series, descriptive case reports, and radiological analyses focusing on sellar or parasellar inflammatory and neoplastic lesions. Systematic reviews and meta-analyses comparing clinical, hormonal, or imaging findings between hypophysitis and NFPMA were also included, provided they presented clearly extractable data. Publications were excluded if they focused exclusively on pediatric populations, on functioning PA, or if they lacked specific diagnostic or imaging information relevant to the objectives of this review.

For each included study, relevant data were carefully extracted regarding patient demographics, clinical presentation, endocrine profile, and radiologic features. Emphasis was placed on elements that might aid in preoperative differentiation between the two entities. These included the presence of CDI, patterns of anterior pituitary hormone deficiency, pituitary stalk morphology and enhancement, gland symmetry, and contrast enhancement characteristics on MRI. Whenever possible, data were cross compared to identify trends, recurring imaging markers, or clusters of clinical and biological features consistently associated with either hypophysitis or NFPMA.

Given the narrative nature of this review, no formal meta-analysis was undertaken. Instead, findings from individual studies were synthesized qualitatively, highlighting points of convergence and divergence among the various reports. Studies with larger patient cohorts or more robust imaging protocols were given greater interpretative weight, while smaller case series and individual reports were used to illustrate particular or emerging aspects of the disease spectrum.

Through this approach, the review aimed not only to summarize the current evidence but also to critically interpret the diagnostic pathways and methodological challenges encountered in previous research. The goal was to build a comprehensive and integrative view of the available data, providing clinicians and radiologists with practical insights into distinguishing these two frequently confounded entities.

## 3. Epidemiological and Anamnestic Criteria

Epidemiological and anamnestic factors provide valuable preliminary clues when differentiating between hypophysitis and NFPMA. Although none of these parameters alone is diagnostic, their constellation may raise or diminish suspicion for one condition over the other, particularly in patients presenting with non-secreting sellar masses of uncertain etiology.

Age represents one of the most reproducible differentiating criteria. Patients with hypophysitis are generally younger than those with NFPMA. Several large case series have reported a mean age of 40 to 45 years for hypophysitis, compared with an average exceeding 50 years for NFPMA [37,38]. This age gap is especially relevant when evaluating a younger patient with an otherwise typical macroadenomatous appearance, as an inflammatory etiology should be strongly considered in such a context. Nevertheless, this distinction must be interpreted cautiously, as late-onset hypophysitis has also been described, particularly in secondary or drug-induced forms [39].

Gender distribution further refines diagnostic suspicion. Hypophysitis displays a clear female predominance, especially in its lymphocytic form which is the most common histopathological subtype. Wright et al. [38] observed that 61% of their patients with hypophysitis were female, whereas Gutenberg et al. [37] and Caturegli et al. [12] reported even higher proportions. In several North African series, this predominance reached 90%, suggesting both hormonal and immunological influences [39,40]. By contrast, NFPMA tends to affect both sexes more evenly, with only a slight female predominance occasionally observed in certain cohorts.

Pregnancy and the postpartum period are particularly relevant in the context of hypophysitis, given their well-established association with lymphocytic inflammation of the pituitary. The immunological changes of late pregnancy and the postpartum period, marked by a rebound in cellular immunity, appear to play a key role in the pathogenesis of this condition [41]. Gutenberg et al. [37] highlighted pregnancy and early postpartum as strong discriminative clinical settings for autoimmune hypophysitis. Similarly, Caturegli et al. [12] reported that more than half of lymphocytic hypophysitis cases occurred within this temporal window. Conversely, this association is virtually absent in NFPMA, whose development is unrelated to reproductive or immune changes.

Another epidemiologic clue lies in the presence of autoimmune comorbidities. The coexistence of autoimmune thyroiditis, type 1 diabetes mellitus, or other organ-specific autoimmune diseases markedly favor a diagnosis of hypophysitis [14,42]. Chiloiro et al. [14] reported autoimmune comorbidities in over 60% of their patients with hypophysitis, whereas such associations are extremely rare in PA. Secondary forms of hypophysitis may also occur in systemic disorders such as sarcoidosis or granulomatosis with polyangiitis, or may be triggered by external factors, including immune checkpoint inhibitors and, more recently, vaccination against SARS-CoV-2 [43,44]. These epidemiological and anamnestic elements, although non-specific, together constitute a strong contextual framework for suspecting hypophysitis in the appropriate clinical setting (Table 1).

## 4. Clinical Presentation

The clinical presentation of hypophysitis and NFPMA often overlaps, as both may manifest through mass effect and varying degrees of anterior pituitary dysfunction. However, subtle differences in symptom frequency, mechanism, and temporal progression can provide meaningful diagnostic orientation [45].

Headache is among the most frequent presenting symptoms in sellar pathology, reported in both entities but with distinct characteristics. In NFPMA, headache is typically more intense and persistent, affecting up to 90% of patients in some series [39]. Its mechanism is primarily mechanical related to dural stretching or diaphragmatic compression caused by the expanding mass [48,49,50]. In hypophysitis, headache is less frequent and is often described as diffuse or pressure-like. It may reflect an inflammatory process rather than true compression, with improvement frequently observed under corticosteroid therapy, a feature that may retrospectively support the diagnosis [38].

Visual disturbances represent another key differentiating element [46]. Chiasmal compression by a macroadenoma typically produces a bitemporal hemianopia, occasionally accompanied by decreased visual acuity or ophthalmoplegia when the tumor extends laterally toward the cavernous sinus [13,51]. These manifestations are highly suggestive of NFPMA and are less commonly observed in hypophysitis. In the study by Wright et al. [38], the absence of visual field defects was one of the features most strongly associated with hypophysitis. Nevertheless, exceptions exist: in diffuse or granulomatous forms of hypophysitis, especially when the inflammation extends beyond the gland into the suprasellar region, chiasmal compression and visual compromise may also occur [12,52]. Hence, while visual symptoms remain a classic hallmark of macroadenomas, they do not fully exclude inflammatory disease [47].

Among all clinical and hormonal clues, CDI stands out as a major discriminating factor. CDI is reported in 18% to 83% of hypophysitis cases, particularly when the inflammatory process involves the infundibulum or posterior lobe [1,9]. Its occurrence is exceedingly rare in NFPMA, where posterior pituitary involvement is exceptional [2,53,54]. The presence of polyuria and polydipsia in the context of a sellar mass should therefore raise strong suspicion of hypophysitis, especially when combined with MRI evidence of stalk thickening or loss of the posterior pituitary bright spot. However, caution is required in interpreting its absence: in some cases, coexisting corticotropic insufficiency may mask diabetes insipidus, rendering its diagnosis more challenging [23,55].

Other features of anterior pituitary dysfunction, such as asthenia, anorexia, weight loss, amenorrhea, or decreased libido, are frequent but non-specific. They result from varying degrees of hypopituitarism and can be encountered in both conditions. In NFPMA, hormonal deficiencies tend to develop progressively as the tumor enlarges, whereas in hypophysitis, they often occur more abruptly and may show partial recovery after corticosteroid therapy. Some viral hypophystis represent an increasingly important etiologic category. They may present with a more acute, severe endocrine picture, often with marked pituitary enlargement. While the core imaging features often hold true, the epidemiological context becomes the paramount diagnostic clue. The reviewed diagnostic scores, developed primarily in cohorts of primary/autoimmune hypophysitis, may require careful interpretation in this setting, as the clinical pre-test probability is altered by the specific medical history.

While no single symptom is pathognomonic, the combination of age, sex, autoimmune background, and specific clinical signs, particularly the presence of CDI and the absence of chiasmal compression, can substantially strengthen the diagnostic suspicion of hypophysitis over NFPMA (Table 2).

## 5. Hormonal Profiles

The hormonal profile is central to distinguishing hypophysitis from NFPMA, because the type and progression of endocrine abnormalities often reflect the underlying disease mechanism. Although both conditions can cause varying degrees of anterior pituitary insufficiency, the pattern and distribution of these deficits usually differ. When interpreted carefully, these differences offer useful clues for diagnosis.

Among all hormonal axes, corticotropic deficiency emerges as the most frequent and the most pathognomonic finding in hypophysitis. Numerous studies have highlighted the predominance of adrenocorticotropic hormone (ACTH) deficiency in these patients [57,58], often presenting as an early and disproportionate feature relative to the modest size of the lesion [59]. This discordance between lesion volume and hormonal impact has long been recognized as a hallmark of inflammatory pituitary disease, reflecting the high vulnerability of corticotrophs to immune-mediated damage. Although Gutenberg et al. [37] and Wright et al. [38] did not formally include this parameter in their diagnostic scores, Ach et al. [39] highlighted its strong diagnostic relevance, often reporting it as the most common hormonal abnormality at presentation. Clinically, corticotropic deficiency may dominate the initial picture, preceding the involvement of other axes and occasionally masking coexisting CDI due to secondary adrenal insufficiency.

In contrast, somatotropic deficiency, mediated by growth hormone (GH) impairment, appears to follow an opposite trend. It is distinctly more prevalent in NFPMA and rare in hypophysitis. This divergence again reflects the structural rather than infiltrative nature of macroadenomatous lesions. GH-secreting cells, situated peripherally in the gland, are more prone to compression from expanding adenomas, whereas in hypophysitis the inflammatory process tends to be diffuse and infiltrative but often spares the somatotropic axis at least in the early stages. Thus, preservation of GH secretion in the presence of other pituitary deficiencies can serve as an indirect clue favoring an inflammatory rather than a neoplastic origin [39].

Hyperprolactinemia represents another biochemical parameter frequently encountered in both conditions, albeit with distinct underlying mechanisms of NFPMA: elevated prolactin levels, reported in up to 38.5% of cases [60], are usually explained by the stalk effect, in which compression of the pituitary stalk interferes with dopaminergic inhibition and leads to disconnection hyperprolactinemia. In hypophysitis, prolactin elevation is also observed, though usually in lower magnitude, and is attributed to inflammatory irritation of the hypothalamo-hypophyseal axis rather than mechanical obstruction. Reported prevalence varies between 20% and 37.5% [61,62], with values rarely exceeding those seen in adenomas, thus offering limited discriminatory value when considered in isolation. Nonetheless, in a broader diagnostic context, mild to moderate hyperprolactinemia accompanying other inflammatory features such as stalk thickening and CDI supports the hypothesis of autoimmune pituitary inflammation.

The overall pattern of hormonal involvement provides a particularly revealing diagnostic narrative. In hypophysitis, deficiencies tend to follow a sequential and hierarchical course, reflecting the gradual progression of immune infiltration. The corticotropic axis is typically affected first, followed by thyrotropic and gonadotropic deficiencies [9,26,27,40]. This ordered involvement mirrors the centripetal spread of the inflammatory process within the gland and contrasts sharply with the often irregular and unpredictable pattern seen in NFPMA. In macroadenomas, hormonal loss depends primarily on the direction and magnitude of mechanical compression rather than an intrinsic cellular vulnerability, leading to heterogeneous and variable endocrine presentations.

The distinct hormonal profiles in hypophysitis and NFPMA arise from fundamentally different disease mechanisms. In hypophysitis, the primary process is immune-mediated infiltration and inflammation, which tends to follow a centripetal pattern within the gland. The corticotroph cells, are often the first and most severely affected, explaining the high prevalence and early onset of ACTH deficiency. Conversely, the somatotroph cells reside more laterally and are relatively spared until late in the inflammatory process, accounting for the lower frequency of GH deficiency. CDI results from direct inflammatory damage to the vasopressin-producing neurons of the hypothalamus or their axons in the infundibulum and posterior pituitary [59].

In contrast, NFPMA causes hormonal deficits primarily through mechanical compression and displacement of the normal pituitary gland and stalk. This results in a more random and mass-dependent pattern of deficiency. Somatotrophs, situated in the lateral wings, are highly vulnerable to lateral compression from an expanding adenoma, leading to the high frequency of GH deficiency [39]. The stalk effect explains the common finding of moderate hyperprolactinemia. ACTH deficiency is less frequent and typically occurs later, as the centrally located corticotrophs are somewhat protected until the mass effect becomes severe or global.

These differences highlight the need to interpret hormonal results together with imaging findings and the clinical picture. Certain combinations are particularly suggestive of hypophysitis, such as a severe corticotropic deficit in the presence of only mild gland enlargement, preservation of the GH axis despite other anterior deficits, or hyperprolactinemia associated with infundibular thickening (Table 3). In contrast, a diffuse or mass-related pattern of hormonal impairment, especially when seen with a large heterogeneous lesion compressing the chiasm, is more indicative of an NFPMA (Table 4).

## 6. Radiological Features

Radiological assessment plays a pivotal role in distinguishing hypophysitis from NFPMA, as both entities may initially present with overlapping sellar enlargement and mass effect. However, when assessed in a systematic and integrated way, several imaging parameters allow for a more refined differentiation based on both morphological and functional criteria (Table 5 and Table 6).

**Figure 1:**
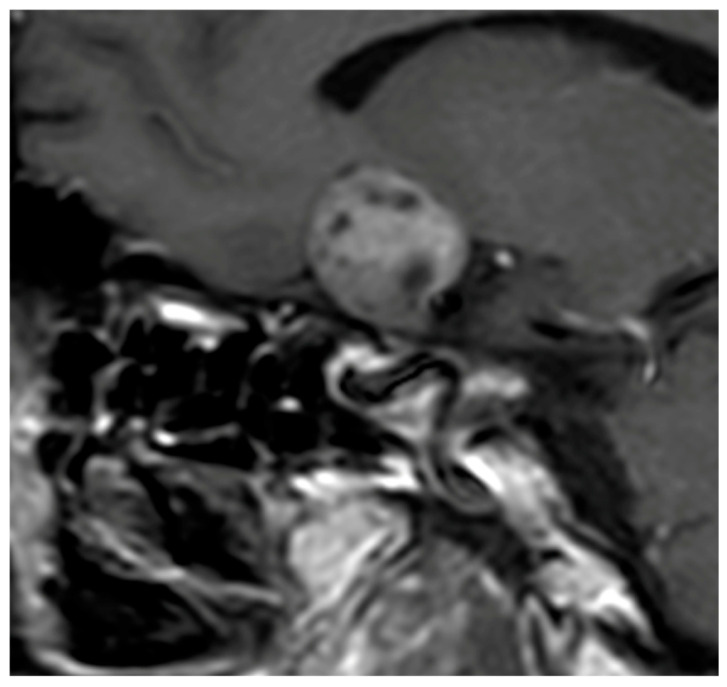
Sagittal T1-weighted image after Gadolinium injection showing a heterogeneous enhancement of the pituitary mass consistent with a NFPMA.

In most series, the tumor volume constitutes one of the most reliable discriminative variables. Hypophysitis typically presents with a moderate and diffuse enlargement of the pituitary gland, in contrast to the more expansive and space-occupying lesions of NFPMA. Gutenberg et al. [37] reported mean volumes around 3 cm^3^ for hypophysitis compared to 10 cm^3^ for NFPMA, identifying a threshold of approximately 7 cm^3^ as particularly useful for distinction. Subsequent studies, including those of Wright et al. [38] and Ach et al. [39], have confirmed this tendency, with mean volumes of 1.45 cm^3^ and 7.16 cm^3,^ respectively, for hypophysitis and NFPMA (Figure 1).

**Figure 2:**
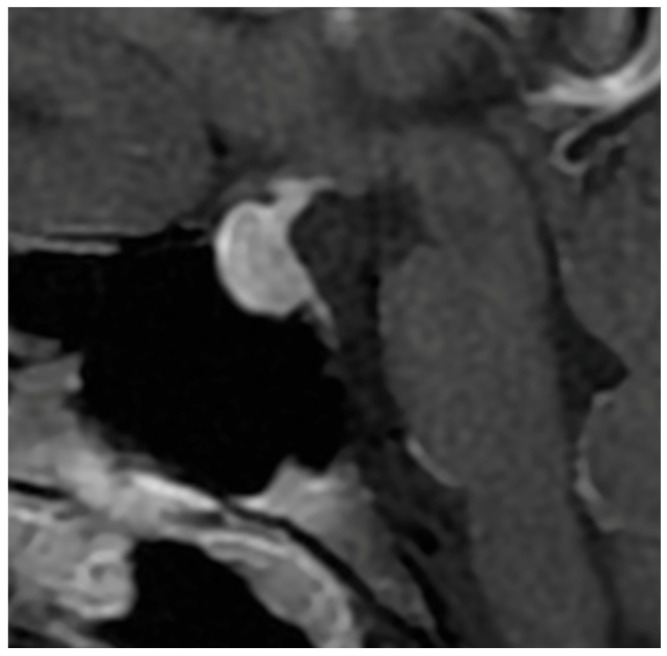
Sagittal T1-weighted image after Gadolinium injection showing a homogeneous enhancement of the pituitary mass in the case of hypophysitis.

Signal characteristics on MRI further reinforce this opposition. Hypophysitis usually exhibits an isointense signal on both T1- and T2-weighted sequences [70,71], indicative of homogeneous inflammatory tissue without necrosis or cystic degeneration. In contrast, NFPMA often show variable signal intensities, with possible T1 hypersignal due to intratumoral hemorrhagic components [37] and frequent T2 hyperintensity related to cystic or necrotic transformation [37,72]. Flanagan et al. [8] and Wright et al. [38] reported T2 hypersignal in only 5 to 8% of hypophysitis, as opposed to 25% in macroadenomas, underscoring the relative uniformity of inflammatory lesions (Figure 2).

**Figure 3:**
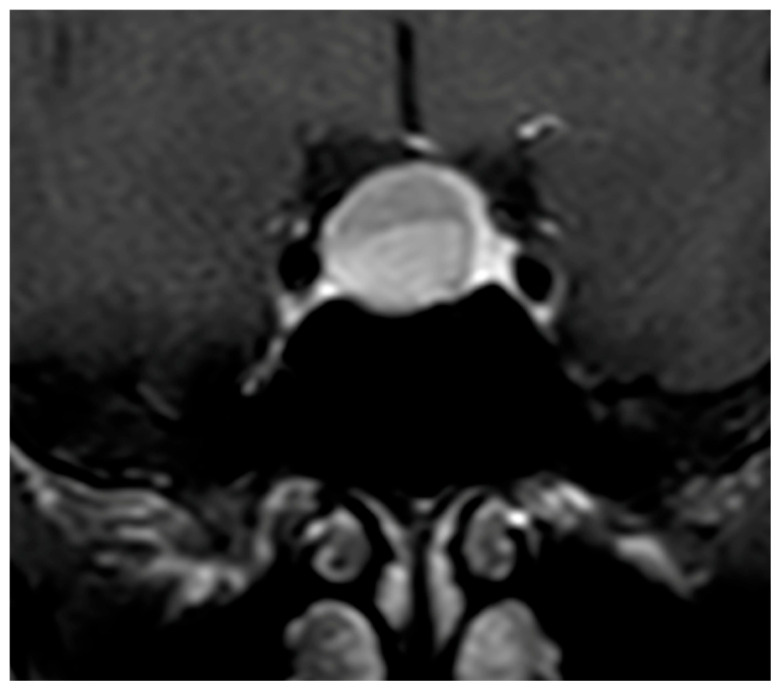
Coronal T1-weighted image after Gadolinium administration showing a NFPMA displacing the normal residual pituitary tissue leftward, which enhances homogeneously and intensely.

The pattern of enhancement after gadolinium injection provides an additional and highly informative contrast between both entities. Hypophysitis typically demonstrates an intense and homogeneous enhancement, reflecting diffuse vascularized inflammation, with reported rates of homogeneity ranging from 70% to 90% [37,73,74]. NFPMA characteristically show a heterogeneous and often delayed enhancement [75,76,77]. The use of early dynamic post-contrast sequences has been advocated by several authors, as it increases sensitivity for detecting subtle differences in enhancement kinetics between infiltrative and neoplastic processes (Figure 3) [78].

Morphologically, symmetry of pituitary enlargement is another hallmark feature suggestive of hypophysitis, while asymmetric growth is more typical of adenomas [39,43].

Particular attention should also be paid to the pituitary stalk, whose morphology often reflects the underlying nature of the lesion. In hypophysitis, thickening of the stalk is frequent and typically smooth, regular, and centered, corresponding to intrinsic inflammatory infiltration [9,38]. In contrast, in NFPMA, any observed stalk thickening tends to be irregular, eccentric, and secondary to extrinsic compression rather than a primary involvement [14,39].

Another characteristic finding is the loss of the posterior pituitary bright spot on T1-weighted images, significantly more frequent in hypophysitis, reflecting neurohypophyseal inflammatory involvement [37,38]. In NFPMA, the bright spot is usually preserved unless markedly compressed or secondarily affected [14,39].

The extent of parasellar invasion and optic chiasm compression further differentiate both entities. These findings are common in NFPMA due to their expansive behavior [9,40], whereas hypophysitis rarely produces significant parasellar extension or chiasmal compression [12,38].

**Figure 4:**
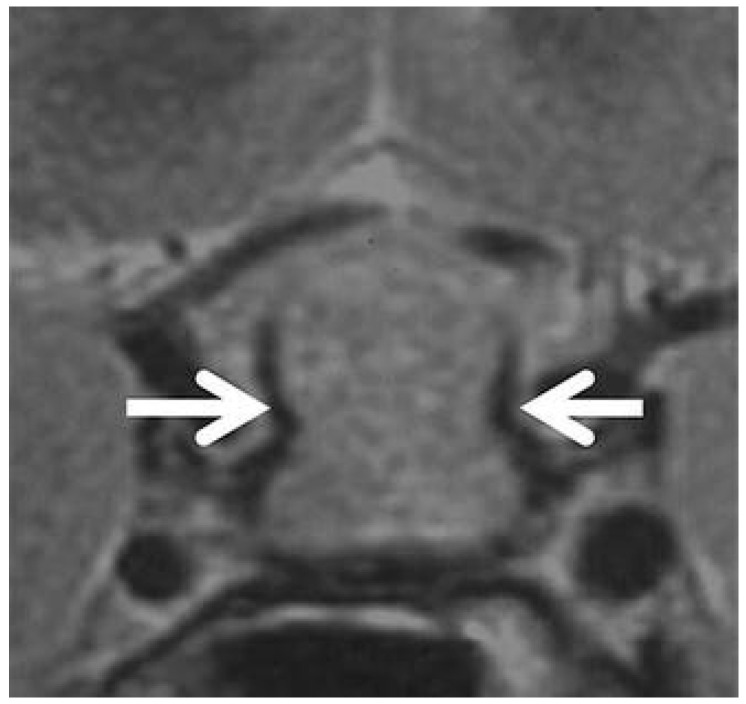
Coronal T2-weighted image showing a peripheral hypointense rim surrounding the parasellar region (white arrows) in a patient with a sellar mass extending superiorly into the suprasellar cistern, consistent with hypophysitis [81].

Several ancillary signs have also been described. Ectopic ADH storage is primarily observed in large NFPMA [79], while the presence of a pituitary pseudocapsule is almost exclusive to adenomas [80]. The dural-tail sign, although occasionally observed [1,3], lacks specificity and has not been retained as a discriminative criterion. Sphenoidal mucosal thickening has been described as more frequent in NFPMA [37,38], though without statistical significance in the study of Ach et al. [39]. Finally, the “parasellar T2 dark sign”, first reported by Nakata et al. [81] and Agarwal et al. [82], corresponds to a peripheral T2 hypointensity highly specific for lymphocytic hypophysitis, possibly related to fibrotic inflammatory tissue (Figure 4). While signs like the parasellar T2 dark sign are promising for their reported specificity, their clinical utility is currently limited by variable reproducibility and a lack of large-scale, multicenter validation. Their presence can support a diagnosis of hypophysitis, but their absence cannot rule it out. This highlights a key limitation of conventional MRI: despite detailed morphologic analysis, significant overlap remains, and no single feature is pathognomonic.

## 7. Diagnostic Scoring Systems

Given the persistent diagnostic overlap between hypophysitis and NFPMA, several authors Given the persistent diagnostic overlap between hypophysitis and NFPMA, several authors have attempted to develop structured diagnostic tools to aid preoperative differentiation. Over the past decade, three predictive scores have emerged, each proposing a combination of clinical, hormonal, and radiologic variables to quantify the likelihood of hypophysitis. Although conceptually similar, these scores differ in their methodological approaches, variable selection, and weighting systems, reflecting the heterogeneity of the studied populations and the evolving understanding of the disease.

### 7.1. Gutenberg et al. Score

The Gutenberg score incorporates a combination of clinical and imaging features to distinguish hypophysitis from NFPMA. It includes age ≤ 30 years, pregnancy-related onset, pituitary volume > 7 cm^3^, moderate or intense contrast enhancement, heterogeneous contrast uptake, asymmetry, loss of the posterior pituitary T1 bright spot, stalk thickening, and sphenoidal mucosal thickening. Clinical parameters such as young age and pregnancy are weighted negatively to favor hypophysitis, whereas imaging features such as increased pituitary volume and heterogeneous enhancement favor NFPMA. Loss of the posterior bright spot and stalk thickening are among the most specific markers for hypophysitis. Strengths of this score include its comprehensiveness and incorporation of specific imaging signs; however, certain parameters such as sphenoidal mucosal thickening and heterogeneous enhancement are nonspecific, and the scoring may overweight clinical over imaging findings. Additionally, it may be less sensitive in atypical demographic groups or in cases with large hypophysitis mimicking macroadenomas (Table 7).

### 7.2. Wright et al. Score

The Wright score is a simpler, four-parameter system including the presence of CDI, absence of cavernous sinus invasion, pituitary stalk thickening, and absence of visual symptoms. Each criterion contributes positively to the probability of hypophysitis, and a score ≥ 3 indicates a high risk, whereas <3 indicates low risk. This score is straightforward to apply and emphasizes highly specific markers such as CDI and stalk thickening. However, its limited number of criteria reduces sensitivity, particularly in atypical presentations, and it does not incorporate pituitary volume or posterior bright spot loss, which are relevant discriminators. It may also overestimate risk in patients with isolated stalk thickening but lacking other hypophysitis features. This score consists of assigning (+2) points if CDI is present, (+2) points if cavernous sinus invasion is absent, (+1) point if infundibular thickening is present and (+1) point if visual symptoms are absent.

### 7.3. Ach et al. Score

This score integrates clinical, endocrine, and imaging features, including female sex, headache, visual symptoms, corticotrope deficiency, pituitary volume < 7 cm^3^, posterior bright spot loss, stalk thickening, optic chiasm compression, and cavernous sinus invasion. Points are assigned to reflect the relative specificity of each feature, with stalk thickening receiving the highest weight (+4.5) and negative points assigned for features more indicative of NFPMA, such as visual symptoms and cavernous sinus invasion. A total score ≥ 0.5 indicates a high risk of hypophysitis. This score offers a more holistic evaluation, balancing clinical, imaging, and hormonal data, and may improve sensitivity for atypical cases. Its main limitations include slightly greater complexity, the use of fractional point values, and the requirement for endocrine evaluation, which may not always be available at the time of imaging. Overall, it represents a balanced approach that captures the most discriminative features identified in both literature and local experience (Table 8).

## 8. Practical Recommendations

Based on the comparative analysis of imaging features and validated scoring systems, several practical recommendations can be proposed for differentiating hypophysitis from NFPMA (Table 9). A comprehensive assessment integrating clinical history [37,39], endocrine evaluation [39], and key imaging features enhances diagnostic accuracy. Among imaging criteria, pituitary stalk thickening and loss of the posterior pituitary bright spot on T1-weighted sequences remain the most specific indicators of hypophysitis [37,39,81,82], whereas cavernous sinus invasion and optic chiasm compression are more suggestive of NFPMA [37,39]. Pituitary volume assessment further aids differentiation, with smaller or moderately enlarged glands favoring hypophysitis and larger masses favoring NFPMA [37,39]. Ancillary signs, including sphenoidal mucosal thickening, dural tail, and parasellar T2 dark sign, may provide supportive information but are less specific and should not be used in isolation [37,38,39,79,80,81,82]. Structured scoring systems can standardize assessment and improve diagnostic confidence: the Gutenberg score offers a comprehensive evaluation but may overweight clinical factors [37], the Wright score highlights highly specific features such as CDI and stalk thickening but may underestimate atypical presentations [38], and the Ach el al. [39] score balances clinical, endocrine, and imaging data, showing promising sensitivity and specificity in local cohorts. MRI protocols should include high-resolution T1-weighted pre- and post-contrast sequences as well as T2-weighted sequences, with careful evaluation of stalk morphology, posterior bright spot, gland volume, enhancement patterns, and adjacent structures. Importantly, imaging scores should guide clinical suspicion but cannot replace histological confirmation when uncertainty persists. Integration of imaging and endocrine findings is particularly valuable for guiding treatment decisions, including the choice between immunosuppressive therapy and surgical intervention. Finally, clinicians should remain aware of the inherent overlap between large hypophysitis and macroadenomas and consider tailored follow-up imaging in equivocal cases to assess dynamic changes typical of inflammatory versus neoplastic processes [37,38,39].

Current MRI-based differentiation, while essential, relies on anatomic and contrast kinetics features that are not disease-specific. Advanced quantitative MRI techniques, such as diffusion-weighted imaging for cellularity assessment, dynamic contrast-enhanced MRI for perfusion/permeability mapping, and magnetic resonance spectroscopy, hold potential as non-invasive biomarkers to distinguish inflammatory from neoplastic tissue. However, their role in this specific differential diagnosis remains investigational, with limited standardized protocols and diagnostic thresholds. Future research should focus on prospective, multi-center studies to validate novel signs and, more importantly, to define the diagnostic accuracy and integrate quantitative parameters from advanced sequences into predictive models or existing scores.

## 9. Conclusions

Differentiating hypophysitis from NFPMA remains a diagnostic challenge due to overlapping clinical and imaging features. Comprehensive assessment integrating patient demographics, clinical presentation, endocrine evaluation, and detailed MRI analysis including pituitary stalk morphology, posterior bright spot, gland volume and cavernous sinus involvement, significantly improves diagnostic accuracy. Structured scoring systems, such as those proposed by Gutenberg, Wright, and Ach et al., provide valuable frameworks to standardize interpretation and support clinical decision-making. While no single feature is entirely pathognomonic, the combination of imaging and endocrine parameters allows for a more confident, non-invasive diagnosis, guiding management strategies that balance the risks and benefits of surgical versus medical intervention. Future research focusing on prospective validation of combined scoring systems and advanced imaging biomarkers is warranted to further refine diagnostic precision and optimize patient outcomes.

## Figures and Tables

**Table 1:** Summary of epidemiological characteristics and key anamnestic data reported in published cases of hypophysitis.

Author, Year	Number of Patients	Mean Age (Years)	Female Gender (%)	Pregnancy and Peripartum (%)	Autoimmune Comorbidities (%)
Ach et al. [39], 2025	16	43.5	93.8	12.5	12.5
Wright et al. [38], 2022	18	42.7	61.1	16.7	22.2
Angelousi et al. [45], 2018	22	31	12	0	36.3
Krishnappa et al. [41], 2022	39	82	39	15.3	-
Khare et al. [24], 2015	24	31.5	87.5	4.1	-
Gutenberg et al. [37], 2009	304	42.3	70	21	-
Imber et al. [46], 2015	21	37.4	62	20	76
Oguz et al. [47], 2020	20	41.5	75	5	10
Wang et al. [48], 2017	50	37	6	16	22
Chiloiro et al. [14], 2019	21	40	81	0	62

**Table 2:** Overview of the clinical presentation of hypophysitis across published series.

Author, Year	Headaches	Visual Disturbances	CDI	Secondary Amenorrhea	Weight Loss	Asthenia
Ach et al. [39], 2025	50%	37.5%	62.5%	25%	43.8%	56.3%
Oguz et al. [47], 2020	63%	37%	32%	66%	-	-
Amereller et al. [56], 2021	38%	17%	38%	-	-	52%
Krishnappa et al. [41], 2022	68%	32%	32%	-	-	38%
Imber et al. [46], 2015	57%	52%	52%	48%	20%	38%
Wright et al. [38], 2022	50%	22.2%	61.1%	-	-	-

CDI: Central Diabetes Insipidus.

**Table 3:** Distribution and frequency of hormonal deficiencies observed in patients with hypophysitis.

Author, Year	ACTH (%)	TSH (%)	LH-FSH (%)	Hyperprolactinemia (%)	GH (%)	CDI (%)
Ach et al. [39], 2025	62.5	31.3	31.3	12.5	18.8	43.7
Oguz et al. [47], 2020	39	61	66	31	21	28
Khare et al. [24], 2015	75	58	50	17	-	16.7
Leung et al. [63], 2004	58	50	91.6	37.5	43	31

**Table 4:** Distribution and frequency of hormonal deficiencies observed in patients with non-functioning pituitary macroadenomas.

Author, Year	GH (%)	LH-FSH (%)	ACTH (%)	TSH (%)	Hyperprolactinemia (%)
Ach et al. [39], 2025	80.4	44.6	17.8	12.5	39.3
Cury et al . [64] , 2009	81.4	63.3	59.9	20.4	38.5
Losa et al. [65], 2008	-	70.7	24.1	25.1	54.3
Ferrante et al. [66] , 2006	-	43.3	26.2	19.6	27.6
Nomikos et al. [67], 2004	-	77.7	31.9	19.6	27.6
Arafah et al. [68], 1986	100	96	81	62	46.1
Wichers-Rother et al. [69], 2004	85	47.3	32.3	27.3	30.6

**Table 5:** Radiological characteristics and imaging patterns described in hypophysitis.

Author, Year	Mean Volume (cm^3^)	T1 Isointense Signal (%)	Cystic T2 Signal (%)	Homogeneous Signal (%)	Intense Enhancement (%)	Homogeneous Enhancement (%)	Symmetry (%)	Thickened Stalk (%)	Loss of Posterior Pituitary T1 Hyperintensity (%)	Cavernous Sinus Invasion (%)	Optic Chiasm Compression (%)	“Dural-tail Sign” (%)	Sphenoid Mucosal Thickening (%)
Ach et al. Score [39], 2023	1.45	100	6.2	93.7	50	93.8	100	62.5	62.5	6.3	0	0	0
Gutenberg et al. [37], 2009	3	69	-	79	73	78	96	68	26	51	60	12	6
Wright et al. [38], 2022	-	-	8.3	-	-	66.7	-	61.1	44.4	11.1	38.5	-	-
Khare et al. [24], 2015	-	-	-	-	-	91.7	91.7	87.5	71.5	8.3	-	8.3	-
Oguz et al. [47], 2020	-	-	-	-	-	23.5	-	17.5	23.5	-	41.2	-	-

**Table 6:** Radiological characteristics and imaging patterns described in non-functioning pituitary macroadenomas.

Author, Year	Mean Volume (cm^3^)	Hemorrhagic T1 Signal (%)	Cystic T2 Signal (%)	Heterogeneous Signal (%)	Moderate Enhancement (%)	Heterogeneous Enhancement (%)	Asymmetry	Loss of Posterior Pituitary T1 Signal (%)	Cavernous Sinus Invasion (%)	Optic Chiasm Compression (%)	“Dural-Tail Sign” (%)	Sphenoid Mucosal Thickening (%)	Thickened Stalk (%)
Ach et al. Score [39], (2023)	7.16	10.7	21	75	98.2	58.9	82.1	25	50	67.9	10.7	44.6	1.7
Gutenberg et al. [37] (2009)	10	41	-	47	55	47	97	3	43	89	12	22	1
Wright et al. [38] (2022)	-	-	-	-	-	-	-	41.1	57.1	63	-	-	7.1

**Table 7:** Gutenberg et al. score analysis.

Variable	Category	β *	OR	95% CI	*p*	Score
Age (years)	≤30	−1.70	0.18	0.029–1.12	0.067	−1
Relation to pregnancy	Yes	−3.71	0.03	0.002–0.42	0.009	−4
Pituitary volume (cm^3^)	≥6	1.83	5.12	1.98–16.28	0.003	2
Gadolinium enhancement type	Medium or high	−1.32	0.59	0.07–0.65	0.011	−1
Gadolinium enhancement features	Heterogeneous	1.41	4.31	1.34–35.8	0.041	1
Symmetry	Asymmetric	2.49	12.1	2.92–49.7	0.007	3
Posterior pituitary bright spot	Lost	−2.42	0.09	0.015–0.51	0.021	−2
Stalk size	Enlarged	−5.34	0.005	0.0004–0.06	<0.001	−5
Mucosal thickening	Present	2.15	8.61	1.25–58.9	0.028	2

Values < 0 suggesting hypophysitis and ≥0 indicating NFPMA. The β * coefficient represents the effect of being in the listed category relative to the reference group.

**Table 8:** Ach et al. score analysis.

Criteria	Points
Female sex	+3
Presence of headaches	−2
Presence of visual disturbances	−2
Corticotropic insufficiency	+1
Pituitary volume < 7 cm^3^	+0.5
Loss of the posterior pituitary bright spot	+0.5
Cavernous sinus invasion	−0.5
Pituitary stalk thickening	+4.5
Optic pathway compression	−2

Values ≥ 0.5 indicates a high risk of hypophysitis.

**Table 9:** Comparison of the diagnostic scores with advantages and limitations.

Feature	Gutenberg et al. Score	Wright et al. Score	Ach et al. Score
Variables Included	Age ≤ 30 years, Pregnancy, Pituitary volume > 7 cm^3^, Enhancement (type/heterogeneity), Symmetry, Posterior bright spot loss, Stalk thickening, Mucosal thickening.	CDI, Cavernous sinus invasion, Infundibular thickening, Visual symptoms.	Female sex, Headache, Visual disturbances, Corticotropic insufficiency, Pituitary volume < 7 cm^3^, Posterior bright spot loss, Cavernous sinus invasion, Stalk thickening, Optic chiasm compression.
Scoring System	Logistic regression coefficients (β) assigned to each variable. Positive sum favors NFPMA; negative sum favors hypophysitis.	Points: CDI (+2), Absent cavernous sinus invasion (+2), Infundibular thickening (+1), Absent visual symptoms (+1).	Points assigned per feature (+3 for female sex, +4.5 for stalk thickening, −2 for visual symptoms, etc.).
Diagnostic Threshold	Total score < 0 suggests hypophysitis; ≥0 suggests NFPMA.	Total score ≥ 3 indicates high risk of hypophysitis; <3 indicates low risk.	Total score ≥ 0.5 indicates high risk of hypophysitis.
Strengths	Comprehensive; quantifies contribution of specific imaging signs.	Simple, rapid bedside/clinic tool; high specificity with CDI.	Integrates clinical, endocrine, and imaging data; higher sensitivity.
Main Limitations	Requires precise volume measurement; includes fewer specific signs (mucosal thickening); complex calculation.	Does not include pituitary volume or posterior bright spot; lower sensitivity for atypical cases.	More complex scoring; requires endocrine workup; fractional points.
Clinical Context	Best for detailed radiological pre-operative planning.	Useful for initial triage based on key clinical/imaging flags.	Optimal when a full multidisciplinary workup is available.

## Data Availability

No new data were created or analyzed in this study.

## Abbreviations

The following abbreviations are used in this manuscript:

ACTH	AdrenoCorticotropic Hormone
ADH	AntiDiuretic Hormone
CDI	Central Diabetes Insipidus
FSH	Follicle-Stimulating Hormone
GH	Growth Hormone
IHC	Immunohistochemistry
LH	Luteinizing Hormone
MeSH	Medical Subject Headings
MRI	Magnetic Resonance Imaging
NFPMA	Non-Functioning Pituitary Macroadenoma
PA	Pituitary Adenomas
SARS-CoV-2	Severe Acute Respiratory Syndrome Coronavirus 2
TSH	Thyroid-Stimulating Hormone
WHO	World Health Organization

## References

[1] Jipa A., Jain V. (2021). Imaging of the sellar and parasellar regions. Clin. Imaging.

[2] Burns J., Hsu K., Shifteh K., Erdfarb A.J. (2019). Neuroendocrine imaging. Adv. Clin. Radiol..

[3] Perosevic M., Jones P.S., Tritos N.A. (2021). Magnetic resonance imaging of the hypothalamo–pituitary region. Handbook of Clinical Neurology, Volume 179—The Human Hypothalamus: Anterior Region.

[4] Ntali G., Wass J.A. (2018). Epidemiology, clinical presentation and diagnosis of non-functioning pituitary adenomas. Pituitary.

[5] Kurihara N., Takahashi S., Higano S., Ikeda H., Mugikura S., Singh L.N., Furuta S., Tamura H., Ishibashi T., Maruoka S. (1998). Hemorrhage in pituitary adenoma: Correlation of MR imaging with operative findings. Eur. Radiol..

[6] Bonneville J.F. (2016). Magnetic resonance imaging of pituitary tumors. Imaging in Endocrine Disorders.

[7] Famini P., Maya M.M., Melmed S. (2011). Pituitary magnetic resonance imaging for sellar and parasellar masses: Ten-year experience in 2598 patients. J. Clin. Endocrinol. Metab..

[8] Flanagan D.E.H., Ibrahim A.E.K., Ellison D.W., Armitage M., Gawne-Cain M., Lees P.D. (2002). Inflammatory hypophysitis—The spectrum of disease. Acta Neurochir..

[9] Langlois F., Varlamov E.V., Fleseriu M. (2022). Hypophysitis, the growing spectrum of a rare pituitary disease. J. Clin. Endocrinol. Metab..

[10] Fontana E., Gaillard R. (2009). Epidemiology of pituitary adenomas: Results of the first Swiss study. Rev. Medicale Suisse.

[11] Fernandez A., Karavitaki N., Wass J.A.H. (2010). Prevalence of pituitary adenomas: A community-based, cross-sectional study in Banbury (Oxfordshire, UK). Clin. Endocrinol..

[12] Caturegli P., Newschaffer C., Olivi A., Pomper M.G., Burger P.C., Rose N.R. (2005). Autoimmune hypophysitis. Endocr. Rev..

[13] Nakamura Y., Okada H., Wada Y., Kajiyama K., Koshiyama H. (2001). Lymphocytic hypophysitis: Its expanding features. J. Endocrinol. Investig..

[14] Chiloiro S., Capoluongo E.D., Tartaglione T., Giampietro A., Bianchi A., Giustina A., Pontecorvi A., De Marinis L. (2019). The changing clinical spectrum of hypophysitis. Trends Endocrinol. Metab..

[15] Tahara S., Osamura R.Y., Hattori Y., Ishisaka E., Inomoto C., Sugihara H., Teramoto A., Morita A. (2023). Concurrent IgG4-related hypophysitis and clinically nonfunctioning gonadotroph pituitary neuroendocrine tumor. BMC Endocr. Disord..

[16] Rawanduzy C.A., Winkler-Schwartz A., Couldwell W.T. (2023). Hypophysitis: Defining histopathologic variants and emerging clinical causative entities. Int. J. Mol. Sci..

[17] Magalhães-Ribeiro C., Furtado A., Baggen Santos R., Mascarenhas L., Costa Correia S., Rocha G., Resende M. (2024). Necrotizing infundibulo-hypophysitis: Case-report and literature review. Br. J. Neurosurg..

[18] Scanarini M., d’Ercole A.J., Rotilio A., Kitromilis N., Mingrino S. (1989). Giant-cell granulomatous hypophysitis: A distinct clinicopathological entity. J. Neurosurg..

[19] Hunn B.H.M., Martin W.G., Simpson S., McLean C.A. (2014). Idiopathic granulomatous hypophysitis: A systematic review of 82 cases. Pituitary.

[20] Deodhare S.S., Bilbao J.M., Kovacs K. (1999). Xanthomatous hypophysitis: A novel entity of obscure etiology. Endocr. Pathol..

[21] Cosman F., Post K.D., Holub D.A., Wardlaw S.L. (1989). Lymphocytic hypophysitis: Report of 3 cases and literature review. Medicine.

[22] Hassoun P., Anayssi E., Salti I. (1985). Granulomatous hypophysitis with hypopituitarism and minimal pituitary enlargement. J. Neurol. Neurosurg. Psychiatry.

[23] Ezzat S., Josse R.G. (1997). Autoimmune hypophysitis. Trends Endocrinol. Metab..

[24] Khare S., Jagtap V.S., Budyal S.R., Kasaliwal R., Kakade H.R., Bukan A., Sankhe S., Lila A.R., Bandgar T., Menon P.S. (2015). Primary (autoimmune) hypophysitis: Single-centre experience. Pituitary.

[25] Gao H., Gu Y.Y., Qiu M.C. (2013). Autoimmune hypophysitis may eventually become empty sella. Neuro Endocrinol. Lett..

[26] Kluczyński Ł., Gilis-Januszewska A., Rogoziński D., Pantofliński J., Hubalewska-Dydejczyk A. (2019). Hypophysitis: New insights into diagnosis and treatment. Endokrynol. Pol..

[27] Joshi M.N., Whitelaw B.C., Carroll P.V. (2018). Hypophysitis: Diagnosis and treatment. Eur. J. Endocrinol..

[28] Ach T., Ben Yahia W., Halloul I., Sghaier F., Atig A. (2023). Neurosarcoidosis-induced hypophysitis mimicking pituitary macroadenoma. Cureus.

[29] Chanson P., Wolf P. (2021). Clinically non-functioning pituitary adenomas. Presse Med..

[30] Lim C.T., Korbonits M.K. (2018). Update on the clinicopathology of pituitary adenomas. Endocr. Pract..

[31] Lopes M.B.S. (2017). The 2017 World Health Organization classification of pituitary tumors. Acta Neuropathol..

[32] Mayson S.E., Snyder P.J. (2014). Silent pituitary adenomas. J. Neurooncol..

[33] Grossman A.B. (2006). The 2004 WHO classification of pituitary tumors: Is it clinically helpful?. Acta Neuropathol..

[34] Yamada S., Asa S.L., Kovacs K., Muller P., Smyth H.S. (1989). Analysis of hormone secretion by clinically nonfunctioning pituitary adenomas. J. Clin. Endocrinol. Metab..

[35] Trouillas J., Girod C., Sassolas G., Claustrat B., Lhéritier M., Dubois M.P., Goutelle A. (1981). Human pituitary gonadotropic adenoma; histological, immunocytochemical, and ultrastructural and hormonal studies in eight cases. J. Pathol..

[36] Galway A.B., Hsueh A.J.W., Daneshdoost L., Zhou M.H., Pavlou S.N., Snyder P.J. (1990). Gonadotroph adenomas in men produce biologically active FSH. J. Clin. Endocrinol. Metab..

[37] Gutenberg A., Larsen J., Lupi I., Rohde V., Caturegli P. (2009). A radiologic score to distinguish autoimmune hypophysitis from nonsecreting pituitary adenoma preoperatively. AJNR Am. J. Neuroradiol..

[38] Wright K., Kim H., Hill T., Lee M., Orillac C., Mogar N., Pacione D., Agrawal N. (2022). Preoperative differentiation of hypophysitis and pituitary adenomas using a novel clinicoradiologic scoring system. Pituitary.

[39] Taieb A., Bouzaouache I., Gasmi A., Ghachem A., Halloul I., Saafi W., Hamza E., Ghada S., Hasni Y., Mhabrech H. (2025). A predictive score incorporating clinical, radiologic, and hormonal parameters to discriminate lymphocytic hypophysitis from non-functioning pituitary macroadenomas. Diagnostics.

[40] De Vries F., Van Furth W.R., Biermasz N.R., Pereira A.M. (2021). Hypophysitis: A comprehensive overview. Presse Med..

[41] Krishnappa B., Shah R., Sarathi V., Lila A.R., Sehemby M.K., Patil V.A., Sankhe S., Shah N., Bandgar T. (2022). Early Pulse Glucocorticoid Therapy and Improved Hormonal Outcomes in Primary Hypophysitis. Neuroendocrinology.

[42] Ach T., El Euch M. (2023). The need to shed light on potential insidious SARS-CoV-2 post-vaccination pituitary lesions. Therapie.

[43] Ach T., Kammoun F., Fekih H.E., Slama N.B.H., Kahloun S., Fredj F.B., Laouani C., Ach K. (2023). Central diabetes insipidus revealing a hypophysitis induced by SARS-CoV-2 vaccine. Therapie.

[44] Taieb A., Asma B.A., Mounira E.E. (2023). Evidences that SARS-CoV-2 Vaccine-Induced apoplexy may not be solely due to ASIA or VITT syndrome’, Commentary on Pituitary apoplexy and COVID-19 vaccination: A case report and literature review. Front. Endocrinol..

[45] Angelousi A., Cohen C., Sosa S., Danilowicz K., Papanastasiou L., Tsoli M., Pal A., Piaditis G., Grossman A., Kaltsas G. (2018). Clinical, Endocrine and Imaging Characteristics of Patients with Primary Hypophysitis. Horm. Metab. Res..

[46] Imber B.S., Lee H.S., Kunwar S., Blevins L.S., Aghi M.K. (2015). Hypophysitis: A single-center case series. Pituitary.

[47] Oguz S.H., Soylemezoglu F., Sendur S.N., Mut M., Oguz K.K., Dagdelen S., Erbas T. (2020). Clinical Characteristics, Management, and Treatment Outcomes of Primary Hypophysitis: A Monocentric Cohort. Horm. Metab. Res..

[48] Wang S., Wang L., Yao Y., Feng F., Yang H., Liang Z., Deng K., You H., Sun J., Xing B. (2017). Primary lymphocytic hypophysitis: Clinical characteristics and treatment of 50 cases in a single centre in China over 18 years. Clin. Endocrinol..

[49] Qin J., Li K., Wang X., Bao Y. (2021). A comparative study of functioning and non-functioning pituitary adenomas. Medicine.

[50] Li M.W.T., Poon S.W.Y., Cheung C., Wong C.K.C., Shing M.M.K., Chow T.T.W., Lee S.L.K., Pang G.S.W., Kwan E.Y.W., Poon G.W.K. (2023). Incidence and Predictors for Oncologic Etiologies in Chinese Children with Pituitary Stalk Thickening. Cancers.

[51] Al-Dahmani K., Mohammad S., Imran F., Theriault C., Doucette S., Zwicker D., Yip C.-E., Clarke D.B., Imran S.A. (2016). Sellar Masses: An Epidemiological Study. Can. J. Neurol. Sci..

[52] Lee M.S., Pless M. (2003). Apoplectic lymphocytic hypophysitis: Case report. J. Neurosurg..

[53] Fukuoka H. (2015). Hypophysitis. Endocrinol. Metab. Clin. N. Am..

[54] Tomkins M., Lawless S., Martin-Grace J., Sherlock M., Thompson C.J. (2022). Diagnosis and Management of Central Diabetes Insipidus in Adults. J. Clin. Endocrinol. Metab..

[55] Raff H. (1987). Glucocorticoid inhibition of neurohypophysial vasopressin secretion. Am. J. Physiol..

[56] Amereller F., Küppers A.M., Schilbach K., Schopohl J., Störmann S. (2021). Clinical Characteristics of Primary Hypophysitis—A Single-Centre Series of 60 Cases. Exp. Clin. Endocrinol. Diabetes.

[57] Paja M., Estrada J., Ojeda A., Ramón y Cajal S., García-Uría J., Lucas T. (1994). Lymphocytic hypophysitis causing hypopituitarism and diabetes insipidus, and associated with autoimmune thyroiditis, in a non-pregnant woman. Postgrad. Med. J..

[58] Escobar-Morreale H., Serrano-Gotarredona J., Varela C. (1994). Isolated adrenocorticotropic hormone deficiency due to probable lymphocytic hypophysitis in a man. J. Endocrinol. Investig..

[59] Guay A.T., Agnello V., Tronic B.C., Gresham D.G., Freidberg S.R. (1987). Lymphocytic hypophysitis in a man. J. Clin. Endocrinol. Metab..

[60] Karavitaki N., Thanabalasingham G., Shore H.C.A., Trifanescu R., Ansorge O., Meston N., Turner H.E., Wass J.A.H. (2006). Do the limits of serum prolactin in disconnection hyperprolactinaemia need re-definition? A study of 226 patients with histologically verified non-functioning pituitary macroadenoma. Clin. Endocrinol..

[61] Prete A., Salvatori R., Feingold K.R., Adler R.A., Ahmed S.F., Anawalt B., Blackman M.R., Chrousos G., Corpas E., de Herder W.W., Dhatariya K., Dungan K. (2000). Hypophysitis. Endotext [Internet].

[62] Tartaglione T., Chiloiro S., Laino M.E., Giampietro A., Gaudino S., Zoli A., Bianchi A., Pontecorvi A., Colosimo C., De Marinis L. (2018). Neuro-radiological features can predict hypopituitarism in primary autoimmune hypophysitis. Pituitary.

[63] Leung G.K.K., Lopes M.B.S., Thorner M.O., Vance M.L., Laws E.R. (2004). Primary hypophysitis: A single-center experience in 16 cases. J. Neurosurg..

[64] Cury MLC de A.R., Fernandes J.C., Machado H.R., Elias L.L., Moreira A.C., de Castro M. (2009). Non-functioning pituitary adenomas: Clinical feature, laboratorial and imaging assessment, therapeutic management and outcome. Arq Bras Endocrinol Metabol..

[65] Losa M., Mortini P., Barzaghi R., Ribotto P., Terreni M.R., Marzoli S.B., Pieralli S., Giovanelli M. (2008). Early results of surgery in patients with nonfunctioning pituitary adenoma and analysis of the risk of tumor recurrence. J. Neurosurg..

[66] Ferrante E., Ferraroni M., Castrignanò T., Menicatti L., Anagni M., Reimondo G., Del Monte P., Bernasconi D., Loli P., Faustini-Fustini M. (2006). Non-functioning pituitary adenoma database: A useful resource to improve the clinical management of pituitary tumors. Eur. J. Endocrinol..

[67] Nomikos P., Ladar C., Fahlbusch R., Buchfelder M. (2004). Impact of primary surgery on pituitary function in patients with non-functioning pituitary adenomas—A study on 721 patients. Acta Neurochir..

[68] Arafah B.M. (1986). Reversible Hypopituitarism in Patients with Large Nonfunctioning Pituitary Adenomas. J. Clin. Endocrinol. Metab..

[69] Wichers-Rother M., Hoven S., Kristof R.A., Bliesener N., Stoffel-Wagner B. (2004). Non-Functioning Pituitary Adenomas: Endocrinological and Clinical Outcome after Transsphenoidal and Transcranial Surgery. Exp. Clin. Endocrinol. Diabetes.

[70] Caranci F., Leone G., Ponsiglione A., Muto M., Tortora F., Muto M., Cirillo S., Brunese L., Cerase A. (2020). Imaging findings in hypophysitis: A review. Radiol. Med..

[71] Bellastella G., Maiorino M.I., Bizzarro A. (2016). Revisitation of autoimmune hypophysitis: Knowledge and uncertainties on pathophysiological and clinical aspects. Pituitary.

[72] Bender B., Honegger J. (2021). Morphological imaging including imaging anatomy. Pituitary Tumors [Internet].

[73] Briet C., Salenave S., Bonneville J.F., Laws E.R., Chanson P. (2015). Pituitary Apoplexy. Endocr. Rev..

[74] Lechan R.M., Toni R., Feingold K.R., Anawalt B., Blackman M.R., Boyce A., Chrousos G., Corpas E., de Herder W.W., Dhatariya K., Dungan K., Hofland J. (2000). Functional Anatomy of the Hypothalamus and Pituitary. Endotext [Internet].

[75] Go J.L., Rajamohan A.G. (2017). Imaging of the Sella and Parasellar Region. Radiol. Clin. N. Am..

[76] Jugenburg M., Kovacs K., Stefaneanu L., Scheithauer B.W. (1995). Vasculature in Nontumorous Hypophyses, Pituitary Adenomas, and Carcinomas: A Quantitative Morphologic Study. Endocr. Pathol..

[77] Miki Y., Matsuo M., Nishizawa S., Kuroda Y., Keyaki A., Makita Y., Kawamura J. (1990). Pituitary adenomas and normal pituitary tissue: Enhancement patterns on gadopentetate-enhanced MR imaging. Radiology.

[78] Miquel L., Testud B., Albarel F., Sahakian N., Cuny T., Graillon T., Brue T., Dufour H., Schleinitz N., Kaplanski G. (2025). Deciphering the Presentation and Etiologies of Hypophysitis Highlights the Need for Repeated Systematical Investigation. J. Clin. Endocrinol. Metab..

[79] Krishnappa B., Shah R., Memon S.S., Diwaker C., Lila A.R., Patil V.A., Shah N.S., Bandgar T.R. (2022). Glucocorticoid therapy as first-line treatment in primary hypophysitis: A systematic review and individual patient data meta-analysis. Endocr. Connect..

[80] Bonneville J.F., Cattin F., Bonneville F. (2009). Imagerie des adénomes hypophysaires. Presse Médicale.

[81] Nakata Y., Sato N., Masumoto T., Mori H., Akai H., Nobusawa H., Adachi Y., Oba H., Ohtomo K. (2010). Parasellar T2 Dark Sign on MR Imaging in Patients with Lymphocytic Hypophysitis. Am. J. Neuroradiol..

[82] Agarwal A., Bathla G. (2020). Parasellar T2 dark sign on magnetic resonance imaging to differentiate lymphocytic hypophysitis from pituitary adenoma. Surg. Neurol. Int..

